# Desmoplastic small round cell tumor of the liver: diagnosing a rare case on liver biopsy

**DOI:** 10.1186/s13000-023-01373-1

**Published:** 2023-07-29

**Authors:** Xiao Feng, Jing Tao, Qiang Zhou, Yi-Dan Qiao, Le-Jian He, Nan Zhang

**Affiliations:** 1 Department of Pathology, Children’s Hospital Affiliated to Zhengzhou University, Henan Children’s Hospital Zhengzhou Children’s Hospital, Zhengzhou, 450018 China; 2 Department of Pathology, Beijing Children’s Hospital, Capital Medical University, National Center for Children’s Health, Beijing, 100045 China

**Keywords:** Desmoplastic small round cell tumor, Liver, EWSR1, WT1

## Abstract

Desmoplastic small round-cell tumors (DSRCT) frequently develop in the retroperitoneum, pelvis, omentum, and mesentery. Here, we present an unusual case of primary DSRCT in the liver. The patient was an 11-year-old boy with multiple solid masses in the liver parenchyma. The tumor in the needle biopsy had a histology revealing a small round cell morphology and desmoplasia. It shows the immunohistochemical features of DSRCT and documentation of EWSR1-WT1 fusion.

A potential diagnostic pitfall is exerted when evaluating liver biopsy, in which DSRCT is a great mimicker and may be easily confused with more common liver malignancies of childhood, such as hepatoblastoma, calcifying nested stromal-epithelial tumor, undifferentiated embryonal sarcoma, and other small round cell tumors, as well as the fibrolamellar variant of hepatocellular carcinoma. This distinction is critical because an accurate therapeutic approach requires a correct diagnosis.

## Introduction

Desmoplastic small round cell tumors (DSRCT) are rare and highly invasive malignant soft tissue tumors that mainly occur in children and young people, with an incidence of 0.0002‰-0.0005‰ [1]. Up to date, fewer than 600 articles have been published in DSRCT literature. Because the clinical features are not obvious, most patients develop metastases when they are found. Histologically, DSRCT has a characteristic morphology of nests of monotonous small round cells within a prominent hypocellular, desmoplastic, and collagenous stroma. Immunophenotypic profiles showed multiple phenotypes with the expression of epithelial, muscle, and neural markers. DSRCT has a specific reciprocal chromosomal translocation, t (11;22) (p13; q12) (*EWSR1-WT1* fusion), which generates a chimeric protein with transcriptional regulatory activity. DSRCT mainly occur in the abdomen, especially in the retroperitoneum, pelvis, peritoneum, or mesentery, and a few cases occur in the parenchyma, including the kidneys and ovaries [2].

Primary DSRCT of the liver is extremely rare, with fewer than 10 reported cases (Table 1). The first case, in which the primary site was the liver, was reported by Chen in a 66-year-old adult female [3–5]. Here, we report a case of primary liver DSRCT in a child. Imaging revealed multiple solid masses in the liver, excluding liver metastases. Small liver biopsies show limited histopathological features that should be differentiated from various benign and malignant tumors to raise awareness of potential diagnostic pitfalls.

**Table 1:** Clinical and pathologic findings of previously published of cases of liver DSRCT

Case	Study		Publication year	Number of cases	Age(yr)	sex	location	Follow up
1	XG Chen et al. [3]	2001		1	66	female	liver	12 months
2	XD Li et al. [4]	2011		1	44	male	liver	NS
3	Zachary E et al. [5]	2018		6	NS	NS	liver	NS

## Materials and methods

The cases were obtained from the Pathology Department of Henan Children’s Hospital and Zhengzhou Children’s Hospital, and all diagnosis and treatment details were obtained from the clinical details of the specimen request forms. The patient signed an informed consent form, and this manuscript was approved by the Ethics Committee of our hospital.

## Results

### Clinical history

**Fig. 1:**
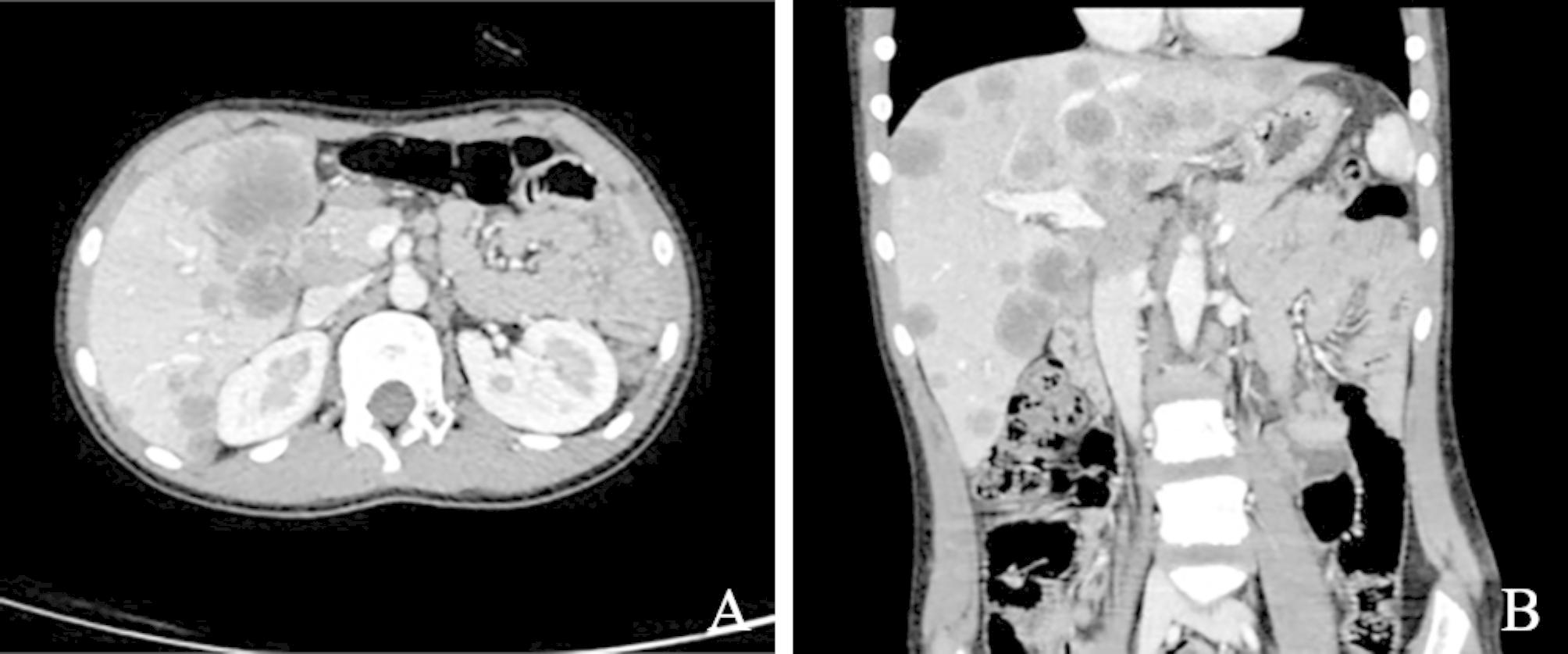
A and **B**, CT scan showed multiple low-density masses in the liver, the largest of which was located in the lower part of the left lobe of the liver (indicated by the white arrow), where A was cross-sectional and B was sagittal

A previously healthy 11-year-old boy presented with complaints of abdominal pain and vomiting and was referred to our hospital because of multiple liver masses discovered by ultrasonography at an outside facility. Laboratory investigations, including blood profiles, liver function tests, and tumor marker levels, were within normal limits. Abdominal computed tomography (CT) showed multiple low-density masses in the liver, the largest of which was located in the lower part of the left inner lobe, with dimension of 44.2 mmx41.5 mmx35.4 mm (Fig. 1A and B). The radiological diagnosis was a lymphoma. No definite pelvic, intraabdominal, or other organ lesions were observed. Tumor puncture biopsy was performed through an ultrasound-guided surgical procedure for pathological diagnosis.

### Pathology

**Fig. 2:**
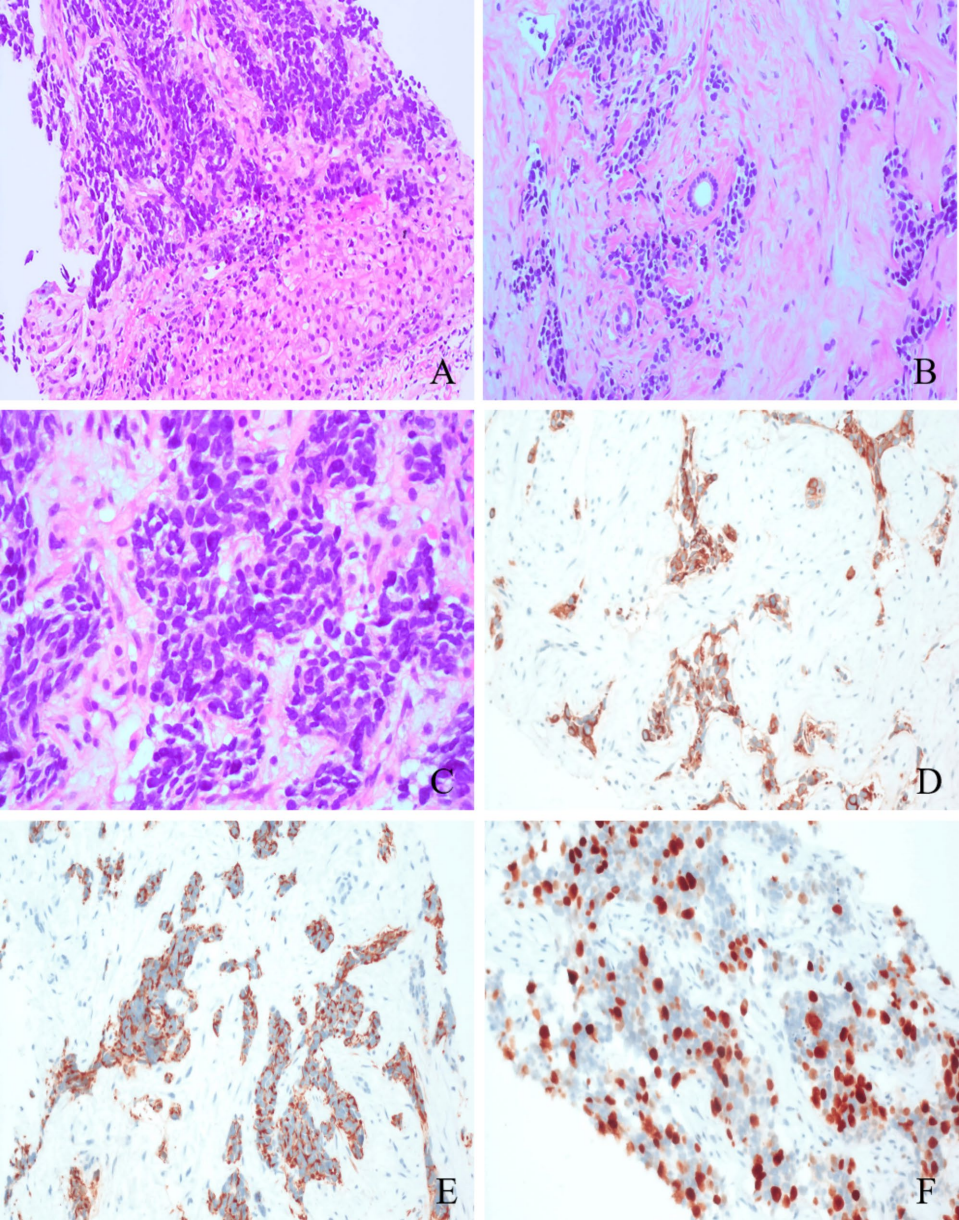
Histology: **A** There are proliferative tumor components in the liver (200×). **B** Tumor cells surround the small bile duct (200×). **C** The nuclei of tumor cells were deeply stained, some of them were vesicular nuclei and no obvious nucleoli, and the cytoplasm was sparse(400×). Immunohistochemistry: **D** AE1/AE3 positive expression (200×). **E** Desmin was expressed in the paranuclear dot-like pattern (200×). **F** Ki-67 index up to 45% positive (200×)

**Fig. 3:**
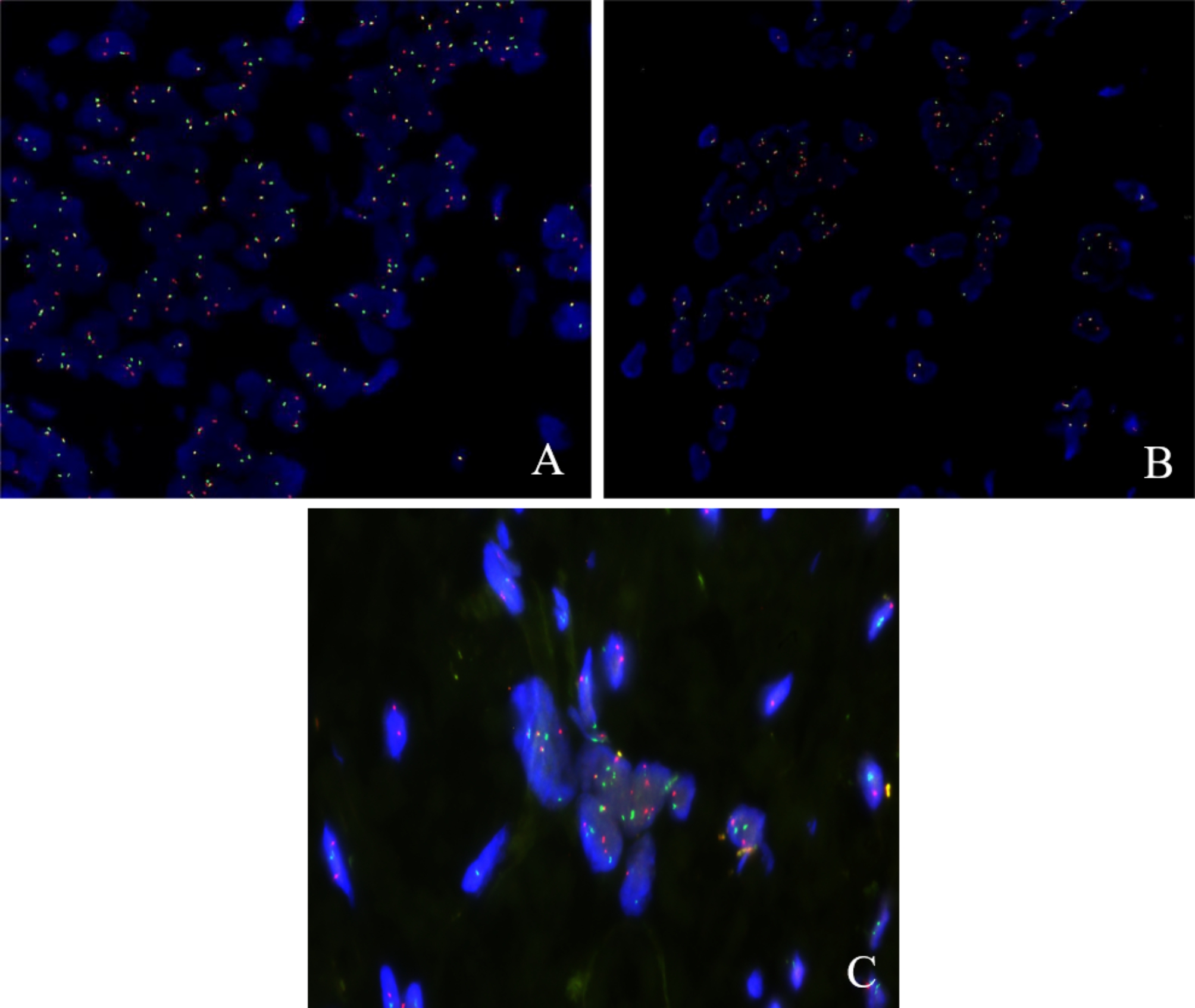
Fluorescence in situ hybridization (FISH): **A** EWSR1 gene was disrupted. **B** WT1 gene was disrupted (EWSR1 and WT1 genes are represented by the red signal (R) and green signal (G). FISH positive cells are one red, one green and one fusion (1R1G1F). 100 cells were counted, 1R1G1F was the typical signal type, and a percentage of more than 15% was considered positive). **C** EWSR1 and WT1 are fused (EWSR1 and WT1 genes were represented by red signal (R) and green signal (G), respectively, and FISH positive cells were one red, one green and two fusions (1R1G2F). 100 cells were counted, 1R1G2F was the typical signal type, and the percentage more than 10% was positive)

Microscopic examination revealed infiltrative neoplastic growth in the liver without capsule formation and around the remnant small bile duct (Fig. 2A and B). The tumor was arranged in well-defined nests embedded in a dense desmoplastic stroma. The neoplastic cells exhibited features of small, round, blue cells with hyperchromatic nuclei, scarce cytoplasm, and inconspicuous nucleoli (Fig. 2C). Some neoplasms contain large amounts of cytoplasm that show vacuolation. There was some karyorrhexis and nuclear shrinkage without mitosis on the biopsy. Most of the stroma was homogeneous and hyalinized, and focally, artificial fissures were observed around the nests of tumor cells, forming pseudo-lacunar structures. We assayed a panel of immunohistochemical markers. Neoplastic cells were immunoreactive to cytokeratin (AE1/AE3 (Fig. 2D), CK7, CAM5.2, and CK19), membrane antigen (EMA), Vimentin and Desmin (Fig. 2E), with smaller numbers expressing TH and synaptophysin. Among the positive profiles, desmin reactivity was observed with solid cytoplasmic and discrete dot-like perinuclear reactivity, and the Ki-67 proliferation index of tumor cells in the hotspots was as high as 45% (Fig. 2F). The assay was negative for the WT1 amino-terminus. However, the FISH assay showed that the tumor cells had *EWSR1* and *WT1* gene divisions, and *EWSR1-WT1* gene fusion was also positive (Fig. 3). Our final diagnosis was a desmoplastic small round-cell tumor of the liver.

### Follow-up

The patient underwent surgical resection, thermal ablation, postoperative adjuvant chemotherapy, and targeted therapy. However, approximately 9 months after the time of diagnosis, the tumor had recurred in the liver, and the child was admitted to the hospital.

## Discussion

DSRCT is a rare neoplasm that accounts for less than 1% of soft tissue sarcomas with extremely aggressive behavior. It was first described by Gerald and Rosai in 1989 and officially named in 1991 [6, 7]. The age of onset of DSRCT is 3–52 years old, about 80% of the patients are 20–30 years old, and the ratio of male to female is 3–5:1 [8, 9]. DSRCT is mainly located in the serosa of abdominal cavity and pelvis, and usually metastasize to the surrounding lymph nodes, liver, lung and so on. Primary DSRCT in parenchymal organs, especially the liver, are rare [10].

Clinical manifestations in patients vary and may be related to the location of the tumor [5, 9]. In addition, it has been reported that CA125 tumor markers have been reported to be upregulated in pelvic DSRCT [11]. The most common imaging finding is multiple, heterogeneous retrovesical or recto-uterine masses without an apparent primary organ of origin, but almost all patients present with a dominant mass. Our patient had no evidence of pelvic, omental, or peritoneal deposits or ascites, which made the primary peritoneal DSRCT less likely to invade the liver.

Histopathological evaluation revealed that the tumor consisted of large nests or small clumps, small ovoid cells, inconspicuous nucleoli, and scant cytoplasm lying in a desmoplastic stroma. Occasional examples of DSRCT have been described to show a wide range of morphological features. Rosette or tubule formation can occur, and focally, tumor cells may have a rhabdoid appearance. Some tumors show focal epithelial differentiation [8, 12]. Immunophenotype of DSRCT is characterized by a polyphenotypic immune profile, including epithelial, neural, and muscle markers, as well as a marked variation in morphological appearances from tumor to tumor and within the same neoplasm [13]. Because of the loss of the amino-terminal of WT1 in the fusion protein, which is induced by gene translocation, DSRCT can be immunoreactive for antibodies selectively directed to the carboxy-terminus of the WT1 protein; with the positive nuclear expression of WT1, antibodies directed against the WT1 amino-terminus are nonreactive [14].

The hallmark characteristic of DSRCT is the *EWSR1-WT1* fusion. The vast majority of patients have the t (11; 22) (p13; q12) translocation, which leads to the fusion of the EWSR1 and WT1 genes on the 11p13 chromosome. The fusion protein of *EWSR1-WT1* may lead to up-regulating transcriptional activation of several genes, such as encoding growth factors and receptors, including *PDGFRα, IGF1R* and *EGFR*, and also transcriptional regulators such as *C-MYC*, *N-MYC* and *PAX2*, which associated with promoting tumor growth and therapeutic resistance [15]. The up-regulation of PDGFRα is a unique event in the development of DSRCT, causing PDGFRα to be responsible for collagenous stromal production and neo-angiogenesis [16], which can explain the microscopic morphology of DSRCT, including stromal proliferation and increased vascular amounts.

Since DSRCT in our case was primary in the liver, it should be distinguished from several liver tumors, including:1) Small cell undifferentiated hepatoblastoma (SCU-HB), which is a special kind of hepatoblastoma in children that is rare and has a poor prognosis. Histologically, SCU-HB cells are small, round, or oval, and slightly larger than lymphocytes, with sparse cytoplasm and inconspicuous nucleoli. However, there was little proliferation of the fibrous stroma, and immunohistochemically, the expression of-catenin, CK19, and the proteins SALL4, GPC3, GS, and MOC31 was unreactive [17]. 2) Fibrolamellar hepatocellular carcinoma (FL-HCC) is a rare liver tumor. The tumor also has a large amount of fibrous connective tissue proliferation, and the tumor cells are divided into nest-like and cord-like cells by fibrous tissue; its growth pattern is very similar to that of DSRCT. The morphology of the cells was larger and the nucleolus was more obvious than that of the DSRCT cells. Immunohistochemical staining revealed positive expression of CK7, CK8, hepatocytes, and DKK-1 [18]. 3) Embryonal tumor of liver (ESL), ESL is common in children and adolescents, and it is rare in clinic, accounting for about 6% of liver tumors in children. Histologically, tumor cells are mainly malignant mesenchymal cells (angiosarcoma-like, bone-, chondrosarcoma-like, and fibroblast-like) and mucous stromal cells. Fibrous connective tissue hyperplasia is also observed. ESL can show the co-expression of CK, Vimentin, Desmin and MSA, similar to the immunohistochemical characteristics of DSRCT. However, ESL had no *EWSR1-WT1* fusion genes [19]. 4) Focal nodular hyperplasia of the liver (FNH), FNH is a benign lesion of the liver, not a real tumor, mostly solitary nodules. The main lesions of typical FNH were nodular, the tumor cells were separated by hyperplastic fibrous tissue, and hyperplasia of thick-walled blood vessels and small bile ducts was observed in the septum; however, there was a lack of nests of small round cells [20]. 5) Calcifying nested stromal‑epithelial tumor (CNSET), CNSET is a rare low-grade malignant tumor of the liver, which is generally multinodular and well defined. Morphologically, the tumor cells were oval or fusiform and arranged in nests. There are two cell types, spindle and epithelioid cells. The immunohistochemical staining was positive for CK, AE1/AE3, and vimentin. They can be distinguished from DSRCT based on cell morphology and immunohistochemical characteristics [21].

In addition, DSRCT should be used to identify other common malignant tumors with small blue round cells, such as extraosseous Ewing’s sarcoma, rhabdomyosarcoma, neuroblastoma, and lymphoblastoma. These tumors can be distinguished from DSRCT in terms of cellular immunohistochemical characteristics and molecular abnormalities.

Currently, there is a lack of effective treatments for patients with DSRCT. Multidisciplinary comprehensive treatment models such as preoperative chemotherapy, postoperative radiotherapy, hot intraperitoneal chemotherapy, and molecular targeted therapy can improve the survival rate of patients [22]. But the prognosis of DSRCT is poor and the survival time is short, with an overall median survival of 2 years and a 5-year survival rate approaching 15% [23, 24].

In summary, primary DSRCT of the liver are extremely rare and can be challenging for core biopsy specimens. Multi-immunophenotypes in DSRCT are distinguished from other neoplasms by the presence of *EWSR1-WT1* translocation. The short survival time of patients with DSRCT reveals the aggressiveness of this disease and the challenge of developing new therapeutic strategies to treat young patients.
